# Resilience in motion: emerging perspectives on stress, substance use and youth

**DOI:** 10.1016/j.ynstr.2026.100783

**Published:** 2026-01-27

**Authors:** P. Sampedro-Piquero

**Affiliations:** Departamento de Psicología Biológica y de la Salud, Facultad de Psicología, Universidad Autónoma de Madrid, Spain

**Keywords:** Alcohol, Exercise, Gender, Interoception, Stress, Resilience

## Abstract

Adolescence and young adulthood are developmental stages characterized by heightened stress sensitivity and limited cognitive control. The identification of the risk factors of alcohol consumption in these stages is crucial for early interventions focused on reducing harmful alcohol use. This review examines how exercise can modulate stress responses, reduce cravings, and preserve cognitive and emotional functioning. In animal models, it has been well described that exercise is able to reduce craving and protect cognitive and affective domains. Translational studies in young people with risky alcohol use (RAU) revealed comparable benefits. Acute aerobic exercise improved executive functions, such as verbal fluency, whereas stretching induced distinct neural oscillatory changes related to emotion regulation. These findings underscore the heterogeneous yet complementary effects of different exercise modalities, suggesting that tailored interventions may optimize outcomes. Future work will incorporate interoceptive measures to clarify the mechanisms linking stress dysregulation and RAU vulnerability, with particular attention to gender-related differences. Collectively, the evidence suggests that aerobic exercise may constitute a promising, feasible, and transdiagnostic intervention that strengthens stress-response systems, reduces craving, and fosters resilience in young people at risk of alcohol misuse, with women showing interoceptive deficits emerging as a particularly vulnerable subgroup.

## Highlights

• Exercise reduced anxiety, craving, and enhanced memory in youths with RAU.
• Virtual reality enabled controlled assessment of stress and craving responses.
• Aerobic exercise and stretching promoted executive and emotional benefits.
• Interoceptive function in women with SUD could be improved by exercise.

## 1 Introduction

Over the past decade, research on exercise as modulator of stress and cognition has expanded rapidly (Chen and Nakagawa, 2023; Nowacka-Chmielewska et al., 2022). Exercise is defined as a planned, structured form of physical activity with the objective of improving or maintaining physical fitness (Caspersen et al., 1985). During the last two decades, interest has steadily increased in both the evidence base and the clinical application of exercise as an adjunctive treatment for individuals with severe mental illnesses, including schizophrenia, bipolar disorder, and major depressive disorder. This growing attention also extends to other conditions, such as anxiety and stress-related disorders, eating disorders and substance use disorders (SUD) (Ashdown-Franks et al., 2020; Solmi et al., 2025). Moreover, it has also been observed that healthy adults who engage in regular exercise exhibit improved mood following exposure to an acute stressor (Childs and de Wit, 2014), however, little is known about its effects in young people, and even less regarding those who use substances such as alcohol. In consequence, this review synthesizes current evidence linking this intervention to resilience and risky substance use, especially alcohol, in youth.

In this context, risky alcohol use (RAU), understood as six or more points for girls and eight or more for boys on the Alcohol Use Disorder Identification Test (AUDIT, Spanish adaptation: Contel Guillamon et al., 1999) (Encuesta sobre Alcohol y Drogas en España (EDADES), 1995–2024), is a pattern of alcohol consumption frequently observed among young people and has been associated to emotional, cognitive and brain alterations (de Goede et al., 2021; Mahedy et al., 2021; Sampedro-Piquero et al., 2024). Multiple therapeutic approaches are currently available to address problematic substance use in young people, including family-based interventions, motivational enhancement therapy, pharmacological treatments, cognitive behavioral therapy, and 12-step programs. However, their effectiveness remains limited, and these interventions may be unsuitable for adolescents and young adults who do not yet perceive their substance use as problematic (Hofmann et al., 2012; Stockings et al., 2016). In this context, several systematic reviews have proposed that exercise-based interventions may offer a promising complementary strategy, as they have been shown to reduce symptoms of anxiety and depression and to enhance overall quality of life (Gür and Can Gür, 2020; Hallgren et al., 2017, 2018; Martínez-Calderon et al., 2023).

Studies conducted in this population have shown that aerobic exercise reduces drug consumption and substance-related measures, such as craving (Buchowski et al., 2011; More et al., 2017). Research involving university students has shown that a single session of moderate-intensity exercise can markedly decrease alcohol craving while enhancing mood and positive affect (Gawor et al., 2021; Herbert et al., 2020). Notably, a recent systematic review by Klamert et al. (2023) found that more than two-thirds of acute exercise studies in young individuals aged 12–25 reported significant improvements in substance-use-related outcomes, whereas only slightly more than half of longer-term interventions demonstrated comparable effects.

Despite these encouraging findings, evidence remains limited regarding the acute effects of exercise on cognitive performance and the associated neural and psychophysiological mechanisms in young people with risky alcohol use (RAU). This gap likely reflects the multifaceted nature of the phenomenon, which is shaped by numerous factors, including the type, intensity, and duration of exercise, as well as age, sex, physical fitness, and severity of alcohol consumption (Ayranci et al., 2025).

## 2 Exercise as modulator of stress and craving: a neurobiological synthesis

Despite this encouraging evidence, the neurobiological mechanisms underpinning the protective impact of exercise on stress-response regulation remain poorly understood, particularly in younger populations with RAU. However, it is known that the preventive potential of exercise for substance use is likely driven by its interaction with the mesolimbic dopamine system, a neural pathway that plays a central role in the initiation of drug use and the development of addictive behaviors (Di Chiara and Imperato, 1988). Notably, exercise reduces attentional bias towards alcohol and other consumption-related cues (Taylor et al., 2013), while also eliciting euphoria and well-being via activation of the mesocorticolimbic reward system, mechanisms that overlap with those triggered by drug abuse (Booher et al., 2020). More recent findings suggest that physical activity facilitates improved regulation of interoceptive signals and homeostatic disturbances linked to craving, thereby enabling individuals with addictive behaviour to recover inhibitory control (Brevers et al., 2024). Additional evidence showed that frequent, moderately intense physical activity elicits transient increases in heart rate, blood pressure, and cortisol (Childs and de Wit, 2014). Repeated activation of the stress-response system may thereby drive adaptive recalibration, resulting in more efficient responses to acute stressors characterized by reduced intensity or faster recovery. However, as a future line of inquiry in this field, it is worth noting that previous studies distinguish between a *threat* and a *challenge* anxiety response (Blascovich and Mendes, 2000; Ensari et al., 2020; Mendes et al., 2001). Both share psychophysiological features such as increased heart rate, yet *threat* responses are associated with peripheral vasoconstriction, whereas *challenge* responses are associated with peripheral vasodilation. Accordingly, high levels of exercise may be more closely related to challenge-type responses and, in turn, may facilitate faster recovery from distress compared to individuals who are less physically active (Bernstein and McNally, 2017). These findings suggest that future research should examine whether exercise-induced enhancements in challenge responses mediate improvements in stress resilience among young people with RAU. In this scenario, stressful events appear to represent risk situations that may precipitate drug use as a strategy to alleviate negative emotional states. An ecologically valid, standardized, and controlled method to recreate such stress-inducing contexts is through virtual reality (VR) techniques. In our recent research project, we employed VR to simulate an elevated plus maze (EPM) scenario, a paradigm widely used in animal models to assess unconditioned anxiety. This immersive environment enables the assessment of behavioural, psychophysiological (cardiac, electrodermal and brain activity), and hormonal (salivary cortisol and alpha-amylase) responses in young participants exposed to the virtual stressor (methodological details in Moreno-Fernández et al., 2023). The VR EPM elicited distinct psychophysiological responses across different measurement points. Electrodermal activity emerged as a sensitive biomarker of stress recovery. Following termination of the stressor, the group with RAU required a longer time to return to baseline compared with the healthy control group. Several studies have considered EDA a reliable peripheral indicator of emotional states and arousal (Gu et al., 2024; Perry Fordson et al., 2022). This points to the need for studies assessing whether EDA-based markers can reliably predict treatment outcomes or relapse risk in young individuals with RAU. In addition, other signaling pathways, such as the opioid, endocannabinoid, and BDNF systems are also thought to play a role, as each contributes to the reinforcing properties of both exercise and substances of abuse (Bi et al., 2024; Psarianos et al., 2023; Sampedro-Piquero and Moreno-Fernández, 2021). Finally, neurophysiological changes have been also documented following a single session of exercise and these findings highlight the extensive range of brain regions affected, including the prefrontal cortex and hippocampus. Among the most notable outcomes of acute exercise are the pronounced shifts in neurochemical activity, encompassing changes in neurotransmitters, metabolites, growth factors, and neuromodulators (Basso and Suzuki, 2017).

Taking together, these broad neurobiological mechanisms clearly illustrate the complex and multifaceted nature of the brain's response to exercise.

## 3 Future directions

Building upon the neurobiological and behavioural evidence reviewed above, several promising avenues for future research emerge. In recent years, a growing line of research has begun to explore the role of interoceptive processing in patients with RAU. The study of interoceptive functioning, that is, the internal perception of bodily states, offers key insights into its relationship with emotional and cognitive domains, as well as with the clinical prognosis of the mental disorder. This approach represents a promising avenue for improving both assessment and therapeutic strategies, particularly in women.

Interoception is a multidimensional construct that encompasses both cognitive and emotional aspects (Critchley and Harrison, 2013; Wiśniewski et al., 2021). Humans can perceive a wide range of internal signals related to physical and emotional well-being, such as energy levels, stress, or mood. This ability is mediated by the interoceptive system, which enables the detection and interpretation of internal bodily signals, including temperature, pain, heart rate, and muscular sensations (Shimohara et al., 2024). Three primary dimensions have been distinguished: *interoceptive accuracy*, defined as the objective ability to detect and track internal signals; *interoceptive sensibility*, which refers to the subjective perception of these signals; and *interoceptive awareness*, understood as the correspondence between objective accuracy and subjective perception, that is, metacognition regarding bodily states (Garfinkel et al., 2015). Alterations in this system have been associated with various mental health conditions, including anxiety, mood, eating, substance use, and psychosomatic disorders, due to its involvement in transdiagnostic dimensions such as emotional regulation, impulsiveness and cognitive deficits (Çöl et al., 2016; Jakubczyk et al., 2021). In this context, recent research has increasingly highlighted the relevance of interoceptive processing in SUD (Brevers et al., 2024; Koob, 2008). For instance, at the physiological level, the long-term effects of alcohol include somatic manifestations such as tremors (Greenfield et al., 2007), hyperactivation of the autonomic nervous system, e.g. arrhythmia, arterial hypertension (Fama et al., 2020), and alterations in body temperature regulation, e.g. abnormal sweating (Price et al., 2019). In consequence, a distorted perception of these signals could negatively impact treatment outcomes.

Accordingly, recent studies have proposed the use of objective interoceptive measures to evaluate craving or withdrawal, focusing on symptoms such as palpitations, sweating, or tremors. Complementary physiological markers, including heart rate variability, galvanic skin response, and accelerometry, have also been suggested as useful tools for this purpose (Billaux et al., 2025). Gender differences further underscore the importance of this line of research. Several studies indicate that, compared with men, women are more likely to consume alcohol as a strategy for regulating negative affect and stress reactivity (Stoica et al., 2021). Carbia et al. (2020) observed that women display greater vulnerability to the emotional effects of alcohol consumption, while Stoica et al. (2021) found that they tend to rely more on frontal control systems for emotional regulation. Moreover, women in treatment for RAU exhibit a higher prevalence of co-morbid mental disorders and interpersonal trauma, factors linked to emotional regulation difficulties and poorer treatment outcomes (Greenfield et al., 2007; Walitzer and Dearing, 2006). In terms of cognitive functioning, chronic and excessive alcohol use in women has been associated with multiple cognitive deficits that overlap with, though are not identical to, those observed in men (Fama et al., 2020; Foster et al., 2014). Although some evidence suggests that women may develop these deficits earlier or with lower cumulative consumption than men, these findings remain inconclusive (Fama et al., 2020). Given the heightened prevalence and earlier onset of emotional and possibly cognitive alterations in women, assessing and training interoceptive skills emerges as a relevant strategy to improve clinical outcomes and reduce the risk of relapse (Price et al., 2019).

Concerning to sex-related physiological mechanisms, estradiol, oxytocin, and prefrontal cortex (PFC) - insula connectivity are closely interconnected in the sex-specific regulation of stress and craving, with estradiol modulating oxytocin levels and function (Quigley et al., 2021). Estradiol plays a critical role in neurobiological regulation, influencing brain region development, cell survival, and stress-response pathways, including the HPA axis, with effects dependent on the estrogen receptor subtype (α or *β*) engaged. Estradiol enhances sensitization in females, which is implicated in craving, telescoping of drug use from intermittent to chronic, and relapse to drug-related cues. Estradiol also enhances the negative components of drug withdrawal associated with the opponent process theory of addiction, but decreases risky decision making, both of which may escalate drug use in women (Quigley et al., 2021). Thus, estradiol is playing an important role in neural processes related to substance use in women, to increase vulnerability. It is also possible that estradiol is acting in men to decrease vulnerability to addiction (Becker et al., 2017; Becker and Chartoff, 2019). On the other hand, estradiol exerts a hormonal influence on the oxytocinergic system, which in turn has protective effects against stress and addiction (Famitafreshi and Karimian, 2021). However, oxytocin's effectiveness varies between sexes, particularly under stress, with evidence suggesting it is less effective in mitigating stress-induced social avoidance in female rodents compared to males (Love, 2018).

Beyond hormonal influences, neural circuit mechanisms also play a critical role in shaping sex-specific responses to stress and craving. The PFC - insula network is central to emotion regulation and responses to salient stimuli, with its connectivity, modulated by sex hormones, interacting with the amygdala to influence stress and craving. Notably, females often show stronger amygdala - PFC connectivity and distinct neural responses to stress and drug-related cues compared to males (Tottenham and Galván, 2016). Hence, insular processing of bodily states increases the imbalance between hypoactivated prefrontal cortex and hyperactivated amygdala-striatal system, favouring immediate substance consumption despite adverse consequences (Noël et al., 2013). Collectively, these findings underscore a complex interplay between hormones, neuropeptides, and neural circuits in shaping sex-specific stress and craving regulation.

Specific strategies to enhance interoceptive awareness, such as mindfulness, biofeedback, and body-centred interventions, may improve emotional and behavioural self-regulation, increase adherence to the treatment, and reduce the rate of relapse. In line with this, brief aerobic exercise programmes could represent a promising future direction (Fig. 1). Such interventions can activate the interoceptive system while improving mood and cognition and are both feasible and easily implemented in clinical contexts, given their capacity to increase physiological activity, e.g. heart rate, and body temperature (Brevers et al., 2024). Preliminary data from our group suggest that, among young people with RAU, acute intense aerobic exercise was associated with reduced interoceptive accuracy (cardiac activity) but increased interoceptive sensibility, assessed with the Borg Rating of Perceived Exertion scale, particularly in girls with emotional dysregulation problems. Regarding this, several studies have observed that interoception plays a key role in the self-regulation of physical activity at different ages (Amaya et al., 2021). These findings support the hypothesis that the interaction between interoceptive awareness and sustained exercise engagement may contribute to lower dropout rates from an exercise program.

**Fig. 1:**
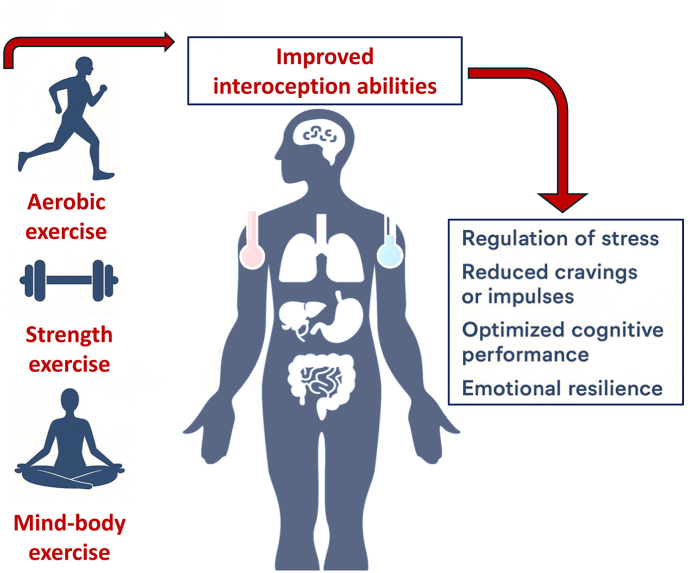
**Exercise as a modulator of interoception.** This figure illustrates how different types of exercise modulate the activity of internal organs, whose accurate perception, e.g. heart rate acceleration, changes in body temperature, intestinal motility, respiratory changes, etc. can influence the stress response, the cognition, or the craving in the context of RAU in youth.

Beyond intervention development, the interoceptive profile could become a valuable clinical marker for prognosis, enabling the identification of more vulnerable patients and supporting the more efficient allocation of clinical resources. Nevertheless, assessing interoceptive abilities in young people with RAU would support the design of more personalized therapeutic interventions. However, this evaluation is a significant challenge, as one of the most used classical measures, the heartbeat-counting task (HCT), has important limitations despite its widespread use in the field of substance abuse (Wiśniewski et al., 2021). Recently, several authors have suggested that this measurement may be influenced by prior knowledge of one's heart rhythm, cognitive strategies, or time estimation (Billaux et al., 2025). Moreover, we cannot ascertain whether the participant is truly sensing or merely counting her heartbeats. To address these limitations, it would be interesting to include the heart rate variability (HRV) and the galvanic skin response (GSR) variables as additional indices that may strengthen the assessment of interoceptive processes in future research (Billaux et al., 2025). From a theoretical standpoint, the study of these psychophysiological variables would contribute empirical evidence to contemporary explanatory models, such as somatic marker theory, which integrates interoceptive processes into the understanding of addictive disorders. Such insights would foster a broader, more integrative view of psychobiological functioning in clinical contexts, highlighting the central role of internal bodily signals in emotional, cognitive, and behavioural regulation. Furthermore, these findings may guide the development of diagnostic tools that complement traditional assessments through the inclusion of objective and subjective interoceptive indicators as part of a comprehensive evaluation of individuals with RAU.

Finally, the specific focus on women addresses the urgent need for gender-sensitive research in the field of SUDs. Exploring the neuropsychological and emotional particularities of this population may inform the design of interventions tailored to the differential characteristics of women with RAU, ultimately promoting more equitable and effective care.

## 4 Conclusions

In conclusion, the findings summarized in this manuscript reinforce the promise of exercise-based and enriched interventions as complementary strategies to address stress-related vulnerability and substance misuse, particularly in young populations. Looking forward, the integration of interoceptive measures represents a critical step towards a more comprehensive understanding of the psychobiological mechanisms underlying RAU, with relevance for women, who often present with heightened emotional vulnerability and comorbidity. By combining behavioural, psychophysiological, and interoceptive markers, future studies may advance the development of gender-sensitive, mechanistically informed, and clinically feasible interventions. In this field, exercise may represent a transdiagnostic approach pending further validation to improve stress-response systems, reduce craving, and promote both cognitive and affective well-being. Together, these avenues highlight the value of embedding lifestyle-based, accessible, and personalized interventions into the treatment and prevention of addictive disorders.

## Statements and declarations

Author declares no conflicts of interest.

## Funding

This study was funded by the Spanish Ministry of Health (Government Delegation for the National Plan on Drugs, code 2022I004 to P.S.-P.) and FEDER/Spanish Ministry of Science and Innovation and the National Research Agency (AEI) (MCIN/AEI/10.13039/501100011033/FEDER, UE, code PID2022-137601OA-I00 to P.S.-P.).

## Declaration of competing interest

The authors declare that they have no known competing financial interests or personal relationships that could have appeared to influence the work reported in this paper.

## Figures and Tables

**figs1:**
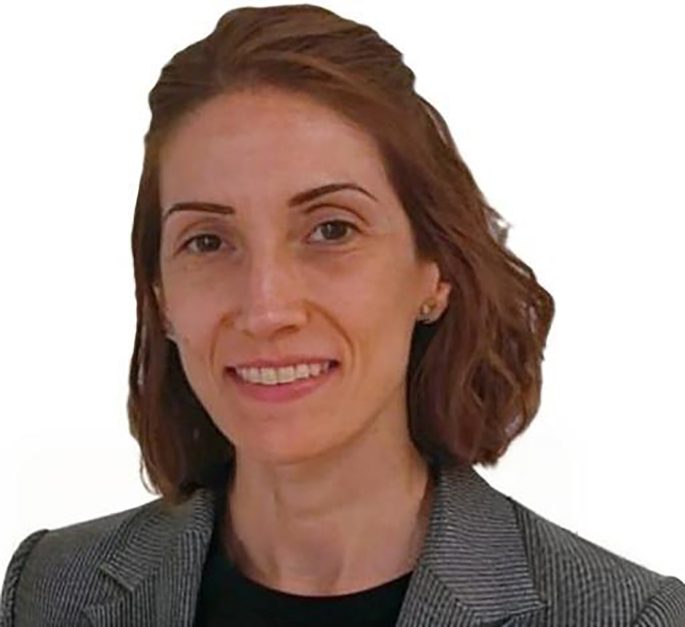


## Data Availability

Data will be made available on request.
